# Infectious causes of chronic inflammatory diseases and cancer.

**DOI:** 10.3201/eid0403.980339

**Published:** 1998

**Authors:** G H Cassell

**Affiliations:** Lilly Research Laboratory, Lilly Corporate Center, Indianapolis, IN 46285, USA. Cassell_Gail_H@Lilly.com

## Abstract

Powerful diagnostic technology, plus the realization that organisms of otherwise unimpressive virulence can produce slowly progressive chronic disease with a wide spectrum of clinical manifestations and disease outcomes, has resulted in the discovery of new infectious agents and new concepts of infectious diseases. The demonstration that final outcome of infection is as much determined by the genetic background of the patient as by the genetic makeup of the infecting agent is indicating that a number of chronic diseases of unknown etiology are caused by one or more infectious agents. One well-known example is the discovery that stomach ulcers are due to Helicobacter pylori. Mycoplasmas may cause chronic lung disease in newborns and chronic asthma in adults, and Chlamydia pneumoniae, a recently identified common cause of acute respiratory infection, has been associated with atherosclerosis. A number of infectious agents that cause or contribute to neoplastic diseases in humans have been documented in the past 6 years. The association and causal role of infectious agents in chronic inflammatory diseases and cancer have major implications for public health, treatment, and prevention.


### Infectious Causes of Chronic Inflammatory Diseases and Cancer

**Gail H. Cassell**
Lilly Research Laboratories, Indianapolis, Indiana, USA

Powerful diagnostic technology, plus the realization that organisms of otherwise unimpressive virulence can produce slowly progressive chronic disease with a wide spectrum of clinical manifestations and disease outcomes, has resulted in the discovery of new infectious agents and new concepts of infectious diseases. The demonstration that final outcome of infection is as much determined by the genetic background of the patient as by the genetic makeup of the infecting agent is indicating that a number of chronic diseases of unknown etiology are caused by one or more infectious agents. One well-known example is the discovery that stomach ulcers are due to *Helicobacter pylori.* Mycoplasmas may cause chronic lung disease in newborns and chronic asthma in adults, and *Chlamydia pneumoniae*, a recently identified common cause of acute respiratory infection, has been associated with atherosclerosis. A number of infectious agents that cause or contribute to neoplastic diseases in humans have been documented in the past 6 years. The association and causal role of infectious agents in chronic inflammatory diseases and cancer have major implications for public health, treatment, and prevention.

The belief that infectious agents are a cause of chronic inflammatory diseases of unknown etiology and of cancer is not new. Approximately 100 years ago, doctors noted a connection between cervical cancer and sexual promiscuity that transcended mere coincidence (1). By 1911, a connection between viruses and cancers in animals had become well established (2). As early as the 1930s, mycoplasmas were proposed as a cause of rheumatoid arthritis in humans, and shortly thereafter, they were proven to be the most common cause of naturally occurring chronic arthritis in animals (3). Proof of causality of cancer and arthritis in humans was more difficult. When searches for infectious agents in cancer and arthritis found none, research began to focus on mechanisms of inflammation, tumorogenesis, and drug discovery. More recently, however, scientists have renewed searches for infectious agents.

Advances in molecular biology and medical devices have revolutionized our ability to detect very low numbers of infectious agents in specimens collected directly from the affected site. HIV has demonstrated the ability of infectious agents to produce slowly progressive, chronic disease with a wide spectrum of clinical manifestations and disease outcomes. Increased understanding of the body's defense mechanisms and the demonstration that final outcome of infection is as much determined by the genetic background of the host as by the genetic makeup of the infecting agent suggest that a number of chronic diseases of unknown etiology may be caused by an infectious agent.

Recent data suggest a role for one or more infectious agents in the following chronic diseases: chronic lung diseases (including asthma), cardiovascular disease, and cancer. Many of the agents implicated are commonly transmissible and are either treatable with existing antibiotics or are potentially treatable with antiviral drugs. Thus, proof of causality in any one of these diseases would have enormous implications for public health, treatment, and prevention. Few areas of research hold greater promise of contributing to our understanding of infectious diseases and the eventual relief of human suffering.

The intent of this paper is not to provide a comprehensive review of chronic inflammatory diseases of unknown etiology and the agents implicated but rather to utilize several models to discuss available data and to illustrate the difficulty in proving causality in chronic inflammatory diseases. The discussion is based

Address for correspondence: Gail H. Cassell, Lilly Research Laboratories, Lilly Corporate Center, Indianapolis, IN 46285, USA; fax: 317-276-1743; e-mail: Cassell_Gail_H@Lilly.com.

upon the following assumptions. Most chronic inflammatory diseases are likely multifactorial. Heredity, environment, and nutrition are critical determinants of disease expression with heredity being the most important.

Theoretically, chronic inflammatory diseases currently of unknown etiology could result from three different types of pathogens: 1) those that are fastidious and previously recognized but because of their fastidiousness or lack of appreciation of their disease-producing potential are not included in the differential diagnosis, and 2) infectious agents previously not recognized that therefore go undetected. Infection with either group can result in misdiagnosis and lack of treatment. Depending upon the biology of the organism and intrinsic and extrinsic factors of the host the organism can persist, resulting in chronic inflammation. The third group of pathogens would be those that elicit an autoimmune response resulting in persistent inflammation without the persistence of the inciting agent. Examples of the first two groups of pathogens will be discussed here using mycoplasmas to typify the first group and *Chlamydia pneumoniae* the second. Finally, recent advances in our understanding of the role of infectious agents in cancer will also be summarized.

### Chronic Lung Diseases

### Murine Chronic Respiratory Disease as a Model System

The difficulty in establishing the infectious etiology of a chronic obstructive lung disease is well illustrated by *Mycoplasma pulmonis* and murine chronic respiratory disease. Proof that *M. pulmonis* can cause this disease took nearly 50 years and required inoculation of germ-free animals (4). Chronic bronchopneumonia in rats was first described in 1915 when this species came into general use for experimental purposes (5). In approximately 1940, a Mycoplasma, later identified as *M. pulmonis*, was recognized as a possible cause (6), but the ubiquity of the organism and its frequent isolation from healthy as well as diseased rats and mice (even from trachea and lungs) soon gave it the reputation of being a commensal with little pathogenic potential. The failure of pure cultures of this organism to consistently produce disease of the lower respiratory tract also precluded its acceptance as the etiologic agent. Only in the early 1970s was *M. pulmonis* alone shown to consistently reproduce all of the characteristic clinical and pathologic features of the natural respiratory disease when inoculated into animals maintained under germ-free conditions (7). Subsequent studies provided explanations for previous difficulties in reproducing the disease.

The respiratory disease caused by *M. pulmonis* is slow to begin and long-lasting. Consequently, the disease has various stages of pathologic lesions and a lack of uniform lesions, even among animals in the same cages (due partly to variables that can affect development of the disease in the lower respiratory tract, such as intracage ammonia produced by bacterial action on soiled bedding, synergy with murine respiratory viruses and other bacterial pathogens, and nutritional factors) (7). However, comparison of animals matched for age, sex, and microbial and environmental factors indicates that heredity is the most critical determinant of susceptibility, lesion character, and disease severity. Susceptibility among animal species and among strains of the same species differ dramatically (8-11).

Intranasal inoculation of *M. pulmonis* produces markedly different lesions in F344 rats and in CD-1 mice, even when the dose is comparable on the basis of lung and body weight. In rats the lesions progress slowly from the upper respiratory tract distally, with alveolar involvement occurring days to months following inoculation, whereas in mice, alveolar lesions develop within hours after infection and are responsible for acute alveolar disease and death within 3 to 5 days. Depending on their genetic background, mice that survive the acute disease develop chronic lung disease characterized by bronchiectasis that persists for up to 18 to 24 months or the lifetime of the animal.

Studies of naturally occurring and experimentally induced disease indicate that *M. pulmonis* also causes a slowly progressing upper genital tract disease in LEW and F344 rats (18). Pups can become infected in utero, at the time of birth due to cervical and vaginal infection of the dams, or via aerosol from dams shortly after birth. Even though the organisms can be shown to colonize the ciliated epithelium of the upper and lower respiratory tracts of pups, microscopic lesions are not detectable for 2 to 6 months depending on the strain of rat. Development of obstructive lung disease can require as long as 12 to 18 months.

Differences in severity and progression of the lung lesions due to *M. pulmonis* in LEW and F344 rats are related to differences in the degree of nonspecific lymphocyte activation in the two strains or an imbalance in regulation of lymphocyte proliferation in LEW rats (12). *M. pulmonis* possesses a potent B cell mitogen, and, in addition, the organism is chemotactic for B cells (13). Interestingly, LEW rats are also more susceptible to other chronic inflammatory diseases, including streptococcal cell-wall induced arthritis, adjuvant-induced arthritis, and allergic encephalomyelitis (12).

### *Ureaplasma urealyticum* as a Cause of Pneumonia in Newborns and Its Association with Chronic Lung Disease (CLD) in Premature Infants

Respiratory dysfunction represents the most common life-threatening problem in premature infants and one of the largest costs of neonatal intensive care (14). Infants weighing less than 1,000 g at birth are more likely than those with greater birth weights to die within the first few days of birth of respiratory-related problems; those who survive are at an increased risk of CLD (15). Approximately 20% of stillborn babies and infants dying within 72 hours of delivery have histologic evidence of pneumonia (16). Yet the true incidence of lower respiratory infection acquired either in utero or at the time of delivery and its contribution to death or development of CLD are unknown. The cause of lower respiratory disease in newborn babies is a diagnostic dilemma because pneumonia in early neonatal life is usually clinically and radiologically indistinguishable from surfactant-deficiency syndrome (17). Furthermore, meaningful cultures from the lung are not easily obtained, whereas cultures of the throat, nasopharynx, and blood are unrevealing or misleading.

#### Pneumonia

The mycoplasma *U. urealyticum*, a common commensal of the lower female genital tract, has recently been shown to cause respiratory disease in newborn infants. Retrospective (18) and prospective (19-21) studies indicate an association of *U. urealyticum* with congenital pneumonia. Case reports also provide evidence that *U. urealyticum* is a cause of pneumonia in newborn infants (22-23). The organism has been isolated from affected lungs in the absence of chlamydiae, viruses, fungi, and bacteria and in the presence of chorioamnionitis and funisitis (40) and has been demonstrated within fetal membranes by immunofluorescence (24) and in lung lesions of newborns by electron and immunofluorescent microscopy (20). The specific immunoglobulin (Ig) M response in several cases of pneumonia in newborns further documents in utero infection (20).

We have found that *U. urealyticum* is the single most common microorganism isolated from endotracheal aspirates of infants who weigh ≤2,500 g and who require supplemental oxygen within the first 24 hours after birth (19). Infants weighing ≤1,000 g and from whom *U. urealyticum* is isolated from the endotracheal aspirate are twice as likely to die as infants of similar birth weight but who are uninfected or infected infants ≥1,000 g. These findings support the hypothesis that only a select group of infants, i.e., those with very low birth weights, is subject to disease due to *U. urealyticum*. This fact may account for the seeming disparities in conclusions regarding the role of *U. urealyticum* in neonatal respiratory disease reached in earlier prospective studies that failed to distinguish this subpopulation at high risk from the whole (25,26).

That endotracheal isolations of *U. urealyticum* represent true infection of the lower respiratory tract is supported by initial isolation of ureaplasmas in numbers exceeding 1,000 CFUs (and sometimes exceeding 10,000 CFUs) and repeated isolations of the organism from tracheal aspirates for weeks and even months in some infants that continue to require mechanical ventilation. That the tracheal isolates are not merely a reflection of contamination from the nasopharynx is supported by the discrepancy in isolation rates between the two sites and recovery of *U. urealyticum* in pure culture from endotracheal aspirates in more than 85% of the infants (19). Concomitant recovery of the organism from blood of up to 26% of those with positive endotracheal aspirates and from cerebrospinal fluid (CSF) of some infants indicate that in some infants the organism is invasive (19). Fourteen percent of *U. urealyticum* endotracheal isolates were from infants born by cesarean section with intact membranes, indicating that in utero transmission occurs rather commonly, at least in premature infants.

In a study of 98 infants, respiratory distress syndrome, the need for assisted ventilation, severe respiratory insufficiency, and death were significantly more common among those infants <34 weeks gestation from whom *U. urealyticum* was recovered from endotracheal aspirates at the time of delivery than among uninfected infants (27). *U. urealyticum* was isolated from 34% of blood cultures and also from four of six CSF samples and in 6 of 11 postmortem brain and lung biopsy pecimens. Eighty-two percent of the ureaplasma isolates were present in pure culture, and 48% of infants born by cesarean section with intact membranes had ureaplasmas isolated from one or more sites.

*U. urealyticum* can induce ciliostasis and mucosal lesions in human fetal tracheal organ cultures (20). Furthermore, we have shown that ureaplasmas isolated from the lungs of human infants with congenital and neonatal pneumonia produce a histologically similar pneumonia in newborn mice (28). Even in this mouse model, age is a critical determinant of disease. Newborn mice are susceptible to colonization of the respiratory tract and development of pneumonia; 14-day-old mice are resistant.

We have shown that endotracheal inoculation of premature baboons (well-established models of premature human infants) with *U. urealyticum* isolated from human infants results in the development of pathologically recognizable pulmonary lesions, including acute bronchiolitis with epithelial ulceration and polymorphonuclear infiltration, which is distinguishable from hyaline membrane disease (29). *U. urealyticum* can be isolated from blood, endotracheal aspirates, and pleural fluid and lung tissue from some of these animals 6 days after infection.

The available evidence provides a strong argument that *U. urealyticum* is a common cause of pneumonia in newborn infants, particularly those born before 34 weeks of gestation. The organism can be isolated from endotracheal aspirates in up to 34% of infants weighing <2,500 g; radiographic evidence of pneumonia is twice as common in these infants as in *U. urealyticum* negative infants (30% vs. 16%, p = .03) (30). Many of these infections develop as a result of in utero exposure. Cases of ureaplasmal pneumonia occur much less frequently in term infants. These findings in infants are consistent with the fact that *U. urealyticum* infection of the chorioamnion is also much more common before 34 weeks of gestation. Lack of transplacental passage of immunoglobulin prior to 32 weeks gestation (31) may partially explain these findings. Experience from mycoplasmal respiratory diseases of animals indicates that preexisting antibody is protective, whereas antibody in the presence of an established infection is rarely effective in elimination of the organism (32).

### CLD in Premature Infants

Some, but not all, studies (33-36) show an association between isolation of *U. urealyticum* from the respiratory tract of newborn infants and the development of CLD (33). Differing results may be obtained because some studies do not limit culture isolation to the affected site (the lower respiratory tract), do not limit their patient population to those at greatest risk (birth weight <1,000 g); or do not limit culture isolation to within 12 hours of delivery, i.e., most likely infected in utero. Several facts suggest that infants who acquire *U. urealyticum* in utero may be at greatest risk for development of CLD. Dyke et al. (34) found *U. urealyticum* in the gastric aspirates of infants ≤1,000 g was associated with a significantly increased risk of CLD in those infants delivered by cesarean section but not in those delivered vaginally. This could result from a longer exposure to *U.urealyticum* as a result of in utero exposure, or it may be a reflection of differences in the virulence of those organisms found only in the cervix versus those that have invasive potential and that can cause an ascending infection from the vagina into the uterus. Along these lines it is of interest that a recent study of 49 preterm infants which included only three infants from whom *U. urealyticum* was recovered within 24 hours of birth found no association with development of CLD (35). The remaining 11 infants were not culturally positive until 48 to 72 hours after birth suggesting that only the three study infants were infected in utero. In another recent study reported by Valencia et al. (36) CLD was found in 26% of *U. urealyticum* infected infants compared to only 4.7% of the noncolonized group. However, these results were not statistically significant possibly because of the small number of patients studied but also possibly because 22% of the patients included did not have cultures performed until between 2 days and 3 months postnatal life.

Isolation of *U. urealyticum* from endotracheal aspirates is not only a risk factor for development of pneumonia but also of precocious dysplastic changes (30). Walsh et al. (38) isolated *U. urealyticum* directly from pleural fluid and tissue collected by open lung biopsy in four of eight infants cultured who had CLD. We (19) continued to recover ureaplasmas from endotracheal aspirates of infants with CLD for months following initial recovery of the organism from endotracheal aspirates within 12 hours of birth.

Available evidence creates a cohesive argument that *U. urealyticum* infection of the lower respiratory tract is a likely risk factor for, and not only associated with, CLD. Because *U. urealyticum* has only recently been suggested as a cause of pneumonia in newborns, it is not routinely sought by most hospital laboratories. Furthermore, the organism is not susceptible to antibiotics used prophylactically in very low birth-weight infants with evidence of respiratory distress. Consequently, the infection, i.e., pneumonia, goes undetected and untreated. Due to the difficulties in diagnosis, most hospital laboratories do not culture for this organism.

The pathophysiology of CLD in premature infants suggests that *U. urealyticum* produces undetected and untreated pneumonia and results in an increased requirement for oxygen and subsequent development of CLD as a result of oxygen toxicity (33,37) or a synergistic effect between the ureaplasmas and hyperoxia. It has been proposed that hyperoxia-induced lung injury contributes to development of CLD by stimulating the proinflammatory cytokine interleukin (IL)-6 (38). *U. urealyticum* may also contribute to the development of CLD by stimulation of proinflammatory cytokines. Infants from whom ureaplasmas are isolated from endotracheal aspirates within the first 24 hours of life are more likely to have neutrophils in their tracheal aspirates on day 2 than are those not colonized (39). Aspirates from colonized infants are also more likely to have class II cytology than those from uncolonized patients at day 2 of life. This may explain why ureaplasma-infected infants respond to dexamethasone therapy (39). These in vivo findings are consistent with the recent demonstration of *U. urealyticum* induction of IL-6 and IL-8 in human neonatal pulmonary fibroblasts even in the absence of hyperoxia (38). Interestingly, together ureaplasmas and hyperoxia resulted in greater stimulation of IL-6 and IL-8 than either alone. This is consistent with the synergism previously demonstrated in vivo between ureaplasmal infection and hyperoxia (37).

Studies in mice also suggest that increased oxygen requirements of very low birth-weight infants might predispose them to lower respiratory tract infection or, alternatively, that *U. urealyticum* infection potentiates oxygen-induced injury (28,37). Exposure to oxidants is known to enhance respiratory disease and death due to *M. pulmonis* respiratory disease in mice (41).

That *U. urealyticum* is a cause of pneumonia in newborns can no longer be questioned. Data provide strong evidence that *U. urealyticum* can be a primary cause or a contributing cofactor in development of CLD in humans, but the data are not definitive. Cohort studies allow follow-up of exposed persons and thus reduce bias, but the designs of these studies cannot rule out the possibility that a third factor associated with *U. urealyticum* is actually the true cause of CLD. However, a randomized trial of exposure to infection in humans is not ethical or practical. Although a randomized trial of antibiotic treatment could provide critical information related to patient management, it would still not bring us closer to proving causality. Even if treatment is found to be efficacious, conclusions about causation will be limited by the fact that the third factor might also be susceptible to the antibiotic chosen. If it is not found to be efficacious, it may be because ureaplasma infection in utero or soon after birth results in irreversible lung damage. Nevertheless, a treatment trial is urgently needed to determine whether appropriate therapy can reduce the incidence of illness and death associated with CLD. First, studies are needed to determine dose and duration of antibiotic therapy and whether currently available antibiotics will even eliminate the organism.

### *Mycoplasma pneumoniae* and *C. pneumoniae* as Causes of Chronic Asthma

Asthma, a CLD characterized by airway obstruction, inflammation, and bronchial hyperresponsiveness to a variety of stimuli, including infections, is a common illness in both pediatric and adult populations. In the United States alone, approximately 12 million people have asthma, resulting in health-care costs of approximately 4.6 billion dollars annually (42). In children, asthma is the most common reason for hospital admissions and school absenteeism (43). Yet the etiology and pathogenesis of this important disease remains poorly defined. Historically, viruses that commonly infect the respiratory tract have been thought to play a role in both provoking asthma exacerbations and in altering responses to other environmental agents that might be involved (44).

*M. pneumoniae* is a common cause of both upper and lower respiratory infection in humans; tracheobronchitis is the most common clinical manifestation (45). Previously thought to cause acute, self-limited disease primarily in persons between 6 and 21 years of age (45), *M. pneumoniae* is now known to be the cause of pneumonia in 20% to 25% of all age groups and to persist in certain persons for weeks to months, resulting in prolonged reduced pulmonary clearance and airway hyperresponsiveness (46,47). Epidemiologic evidence links mycoplasma infection with asthma exacerbation and possibly with the pathogenesis of asthma (47-50).

While *M. pneumoniae* has been associated with exacerbations of asthma, its role in sustaining chronic asthma or in initiating exacerbation is unknown. However, the proven role of mycoplasmas in similar chronic respiratory diseases of numerous animal species, including *M. pulmonis* in rodents, suggests that careful, systematic studies should be undertaken in humans (45).

*C. pneumoniae*, the most recently described *Chlamydia* species, has been associated with a wide range of respiratory tract illnesses, from pharyngitis to pneumonia with empyema (51). *C. pneumoniae* has been isolated from 15% to 20% of adults and children with community-acquired pneumonia (51). On the basis of serologic results only, *C. pneumoniae* has been associated with acute exacerbations of asthma in adults (52); on the basis of nasopharyngeal cultures, it has been associated with asthma in children (53). In both children and adults, the organism persists for months in the upper respiratory tract of patients with wheezing (54).

If *M. pneumoniae*, or for that matter any infectious agent, is a causal factor in initiating and sustaining asthma in certain persons, the agent should be present and persistent in the lungs of some persons with stable, chronic asthma. We have recently undertaken a study to determine if *M. pneumoniae* can be detected in the lungs of adults with stable, chronic asthma versus asymptomatic controls (55). To facilitate interpretation of results, we also evaluated the presence of other fastidious infectious agents that have previously been implicated in the pathogenesis of asthma, including the seven common respiratory viruses (44) and *C. pneumoniae* (56,57).

*M. pneumoniae* was detected by PCR in 10 of 18 asthma patients and 1 of 11 controls ($p = 0.02$). All patients were culture, EIA, and serologically negative for *M. pneumoniae*. All PCR and cultures were negative for *C. pneumoniae* and all EIAs for respiratory viruses were negative. Nine persons with asthma and one control exhibited positive serology for *C. pneumoniae* ($p = 0.05$). For *C. pneumoniae*, the lack of correlation between serologic results and culture and PCR was not unexpected. We have seen discordance between culture and serologic results in patients with community-acquired pneumonia (58,59), but in these cases more patients were culture positive than seropositive. Thus, the culture methods we used in the study have previously been shown to be valid.

Our failure to culture *M. pneumoniae* might be explained by its extreme fastidiousness or its low numbers. Culture is the least sensitive of the methods used in this study for detection of *M. pneumoniae*. However, the culture methods we used in this study we also used to evaluate more than 2,000 respiratory specimens during the same period in patients with radiographically confirmed, community-acquired pneumonia. These methods have resulted in recovery of *M. pneumoniae* by culture in up to 17% of patients (G. Cassell, et al., unpub. obs.; 58,59).

Recent studies indicate that some other mycoplasma species of human origin may be able to survive intracellularly in chronic infections of cell cultures (60). Likewise, some strains of *M. hyorhinis*, the etiologic agent of chronic respiratory disease of swine, can become so adapted to growth in the presence of cells that it is no longer cultivable on artificial media (61). If this occurs in vivo, organisms like *M. pneumoniae* could be difficult if not impossible to recover by culture.

In the absence of other known respiratory pathogens in other patient populations, the presence of *M. pneumoniae* can be detected longer by PCR than by either culture or serologic test. Guinea pigs experimentally infected with *M. pneumoniae* become chronically infected as detected by PCR for up to 200 days but are culture negative by 70 days. Also by 70 days, antibody levels become negative (62). Thus, patients with asthma appear chronically infected with *M. pneumoniae*, despite negative culture results, because they are PCR positive. That positive PCR results truly reflect involvement of the lower respiratory tract by *M. pneumoniae* is supported by the fact that 9 of the 10 *M. pneumoniae*-positive patients were positive in the bronchoalveolar lavage (BAL), bronchial biopsies, or bronchial brush specimens. Furthermore, the organism was detected in the nasopharynx or the throat of only five of the nine asthma-positive patients, thus indicating that detection in the lower tract was not merely due to contamination by organisms from the upper tract during sample collection. More importantly, a significant number of persons with asthma were positive in the lower respiratory tract on repeat sampling (2 to 4 months between samples), thus indicating persistent colonization. By cloning and sequencing the PCR product in BAL from several representative patients, we demonstrated 100% sequence homology with *M. pneumoniae.* Use of multiple primer pairs as well as confirmation of PCR findings in two different laboratories also attests to the validity of the PCR results. Our failure to detect *M. pneumoniae* in specimens from age-matched control patients as well as in specimens from 100 asymptomatic children using the same PCR methods further verifies the specificity of our PCR methods and argues that finding *M. pneumoniae* in persons with chronic asthma does not merely reflect a carrier state.

We have previously noted the lack of antibody response to *M. pneumoniae* in both pediatric and adult populations with community-acquired pneumonia (G. Cassell et al, unpub. obs.; 58,59,63). Study results indicate that a subset of infected persons do not mount an antibody response, perhaps due to genetic differences. Lack of antibody may in fact contribute to the organism's persistence. The immunomodulatory properties of *M. pneumoniae* (12) also could facilitate the organism's persistence.

Recent studies indicate that *M. pneumoniae* respiratory disease is often misdiagnosed and inappropriately treated, which would also contribute to persistence. Admitting physicians chose other pathogens as the most likely agents in 46% of the cases subsequently documented as *M. pneumoniae* infections (64). Even upon correct diagnosis, at least 10% of the patients did not receive appropriate antibiotics during their hospitalization.

In summary, we have demonstrated that persons with chronic asthma, but not healthy persons, exhibit evidence of *M. pneumoniae* colonization of the lower airways. Like several other investigators (56,57), we found more persons with asthma than control subjects had serologic evidence of *C. pneumonia* infection. Further study is needed to determine if these findings are an epiphenomenon or, as we expect, a pathogenic mechanism in asthma. If the latter is correct, greater evaluation of the process involved is needed to further our understanding of the pathogenesis and treatment of asthma.

### Role of *C. pneumoniae* in Atherosclerosis

Infection was proposed as a cause of atherosclerosis by Sir William Osler and others at the beginning of the century (65). However, it was not until the 1970s that experimental infection of germ-free chickens with an avian herpesvirus was found to produce arterial disease that resembled human atherosclerosis (66). Associations have since been reported of human coronary heart disease with certain gram-negative bacteria (i.e., *Helicobacter pylori* and *C. pneumoniae)* (67,68), with certain herpesviruses (especially cytomegalovirus) (69), and with clinical markers of chronic dental infection (70). Rather than an exhaustive evaluation of each of these purported associations, it seems reasonable to focus on the respiratory pathogen, *C. pneumoniae,* for which the evidence seems strongest.

*C. pneumoniae*, like *M. pneumoniae*, is a common cause of community-acquired pneumonia (70,71). *C. pneumoniae* infects more than 50% of people at some point in their lives (51,71). It can often go undiagnosed and improperly treated because again it is fastidious and diagnostic methods are not routinely available. Even in the best reference laboratories, diagnosis can be a challenge (71). It, like *M. pneumoniae,* is also thought to play a role in acute asthma and chronic bronchitis (52) as well as to cause extrapulmonary manifestations (51,71). It, like *M. pneumoniae*, can also result in persistent infection following acute respiratory disease (54).

Eighteen seroepidemiologic studies evaluated the association of *C. pneumoniae* infection and cardiovascular disease (67). Most found at least twofold or larger odds ratios; some reported increasing odds ratios with increasing antibody titers. The general consistency of their findings in a total of 2,700 cases supports the existence of some real association between *C. pneumoniae* and coronary heart disease because the studies were done in different populations, used different criteria for cases, adjusted for potential confounders to differing degrees, and were, therefore, prone to different biases. While diagnosis by serology has its limitations, *C. pneumoniae* has been demonstrated by a variety of laboratory techniques (including culture, PCR, electron microscopy, and immunocytochemistry) in the atherosclerotic lesions of coronary arteries, carotid arteries, aorta, smaller cerebral vessels, and larger peripheral arteries (72-78). In the more than 13 published studies of *C. pneumoniae* in human pathology samples (67), chlamydiae were present in 257 (52%) of 495 atheromatous lesions but in only 6 (5%) of 118 control samples of arterial tissue, yielding a weighted odds ratio of about 10 (95% confidence interval 5-22). It seems unlikely that sampling biases can entirely account for this extreme difference between case and control tissue.

*C. pneumoniae*, an obligatory intracellular bacterium capable of causing persistent infection and multiplying in endothelial and smooth muscle cells and macrophages (79), can also be disseminated by macrophages (80). Hence, some have argued that macrophages may ingest *C. pneumoniae* in the lung or elsewhere before migrating to atheromatous lesions, in which case it may only be a bystander. However, in two different rabbit models, atherosclerotic changes develop only after infection with *C. pneumoniae* (81,82). The organism by itself induces the production of cytokines (83) and adhesion molecules (84), and it possesses an endotoxin (85) capable of modulating the host inflammatory response. Thus, the biologic properties of *C. pneumoniae* make it a logical candidate for triggering the chronic inflammation found in atherosclerosis (82).

Finally, some studies have found rising or elevated levels of antibodies to *C. pneumoniae* in some males during the months just preceding a heart attack (86). Recent studies indicate that antibiotics given during or after a first heart attack may decrease the risk of a second cardiac problem (86-88). This finding also raises the possibility that antibiotics may have a role in the treatment of cardiovascular illnesses; that could be especially beneficial in developing countries where traditional treatments like angioplasty are expensive.

Some have proposed additional large-scale antibiotic treatment trials in an attempt to further prove causality. Several major issues need to be resolved first. Ideally, one should treat patients with documented *C. pneumoniae* infection; however, reliable diagnostic methods and treatment protocols are lacking (71). Because most available antibiotics are bacteriostatic, not bacteriocidal, some patients may remain infected up to 11 months after treatment. Even if these issues could be resolved, antibiotic treatment trials will not prove causality, just as is the case with *U. urealyticum* and CLD of prematurity or *M. pneumoniae* and chronic asthma. The nonantimicrobial effects may also influence the outcome of such studies. For example, tetracyclines can inhibit metalloproteinases, which may contribute to acute coronary syndromes (89). Some macrolides have antiinflammatory effects (90-93). Moreover, antibiotics are not selective, thus making it impossible to determine the effects of treatment upon *C. pneumoniae* versus other potential culprits, e.g., *H. pylori,* which is also susceptible to tetracyclines and macrolides. However, if antibiotic treatment could reduce atherosclerotic events, the public health implications could be enormous.

### Causal Role of Viruses and Bacteria in Cancer

Early in this century, Peyton Rous (2) established beyond doubt that cancer can be caused by an infectious agent in chickens. Since then, evidence has accumulated that other viruses cause cancer in a number of different animal species (94). A growing body of research suggests that a number of viruses, bacteria, and parasites cause cancer in humans, thus providing new possibilities for treatment and prevention of cancer (94). In 1997, the World Health Organization estimated that up to 84% of cases of some cancers are attributable to viruses, bacteria, and parasites and that more than 1.5 million (15%) new cases each year could be avoided by preventing the infectious disease associated with them (95).

*H. pylori,* found in the stomachs of a third of all adults in the United States, causes inflammation of the mucous membrane of the stomach (96). In 20% of infected persons, *H. pylori* induces gastric ulcers (96). Peptic ulcer disease, a chronic inflammatory condition of the stomach and duodenum, affects as many as 10% of people in the United States at some time in their lives. In the early 20th century, pathogenesis was believed related to stress and dietary factors. Thus treatment focused on bed rest and bland food. Later, gastric ulcers were believed to be caused by the injurious effects of digestive secretions. Following the identification of the histamine receptor that appeared to be the principal mediator of gastric acid secretion, antagonists of this receptor were used for therapy for peptic ulcer disease. In 1982, *H. pylori* was first isolated from the human stomach, but it was not until one decade later and after Marshall ingested pure cultures of the organism that causality was accepted by the medical and scientific community (97).

In 1994, the International Agency for Research on Cancer concluded that infection of humans with *H. pylori* is causally associated with the risk of developing adenocarcinoma of the stomach (98), one of the most common malignancies in the world, although relatively uncommon in the United States (24,000 new cases and 14,000 deaths per year). However, also in 1994, a Consensus Panel of the National Institutes of Health (NIH) concluded that available evidence was insufficient to recommend eradication of *H. pylori* for the purpose of preventing gastric cancer (99). The NIH conclusion was based upon the existence of clear examples of disparity in the epidemiology of the two diseases. Gastric cancer is more common in males than in females, whereas the rates of *H. pylori* infection are not different for the two genders. Some populations are reported to have a high rate of *H. pylori* infection but low rates of gastric cancer. Gastric cancer occurs in some persons with no evidence of *H. pylori* infection, and in the United States, fewer than 1% of *H. pylori*–infected persons will ever develop gastric cancer. The strongest evidence that *H. pylori* is associated with gastric cancer comes from three prospective studies that indicate that *H. pylori*–infected persons have a significantly increased rate of gastric cancer (96,98).

Only some retrospective serologic studies have shown an association. These disparities indicate that factors other than *H. pylori* infection are also important in gastric cancer risk. It is possible that only some strains of *H. pylori* are involved in the carcinogenic process. For example, infection with *H. pylori* strains possessing the cagA virulence factor is associated with an increased risk of developing adenocarcinoma of the stomach (100,101).

*H. pylori* is also associated with two less common forms of cancer, non-Hodgkin lymphoma and mucosa-associated lymphoid tissue lymphomas of the stomach (96). These types of lymphomas in the stomach only arise in the setting of *H. pylori* inflammation. In 70% of *H. pylori*–infected patients with lymphoma, treatment with appropriate antibiotics leads to regression (96). This finding not only suggests a causal role but that treatment of a bacterial infection can actually result in regression of cancer.

Another landmark study, published in June, 1997, shows that a 12-year nationwide vaccination program against hepatitis B virus in Taiwan resulted in a significant reduction in the number of cases of childhood liver cancer (102). The role of chronic infection with hepatitis B virus in the etiology of hepatocellular carcinoma is well established (103,104). Yet this is the first evidence that prevention of a viral infection is also effective against cancer. The implications are profound. Hepatitis B infection causes some 316,000 cases of liver cancer (60% of all liver cancer) a year worldwide (103,104). While hepatitis C causes a further 118,000 cases (22% of all cases) a year (103,104), some cases result from infections with both viruses (104).

The infectious origin of carcinoma of the cervix has long been suspected, because known risk factors for the disease are linked to sexual activity (105). Recent evidence indicates that human papillomavirus (HPV) types 16 and 18 are definitely carcinogenic in humans (94,105). Types 31 and 33 are classified as probably carcinogenic (94,105). In the United States, HPVs, are associated with 82% of the 15,000 cases and 4,600 deaths due to cervical cancer each year. They are also associated with more than a million precancerous lesions of varying severity. The combined direct medical costs due to HPV are approximately 1.3 billion dollars per year in the United States alone. Thus, effective therapy and vaccines would have a major impact.

The pathogenic mechanisms by which infectious agents cause cancer have not been resolved but they appear to be diverse. In cervical cancer, there seems to be a clear role for HPV-encoded genes in tumor cell growth (106). In addition to stimulation of cell proliferation, inactivation of tumor suppressor genes, such as *p53* may be a common pathway leading to malignancy in HPV and hepatitis B virus (106,107). In the case of other viruses and *H. pylori*, active oxygen and nitrogenic species generated by inflammatory cells may cause DNA damage, induce apoptosis, and modulate enzyme activities and gene expression (94,108).

### Future Research Opportunities

The basic biology of agents implicated in chronic diseases and cancer, in contrast to many other infectious agents, is relatively unknown. With rare exception, the means by which pathogens suppress, subvert, or evade host defenses and establish chronic or latent infection have received little attention. Few areas of basic research compared with microbial latency hold greater promise of substantially contributing to our understanding of infectious diseases and the eventual relief of human suffering (109). Given that the diseases discussed are among the most common in the world, even if only some cases are proven to be of infectious origin and effective therapies or vaccines can be developed, the impact on reducing health-care costs would be substantial. Thus, further research to clarify the etiologic agents and pathogenic mechanisms involved in chronic diseases and cancer should be given the highest priority.

To address the potential role of infectious agents in chronic diseases requires a new research paradigm compared to that which most investigators and funding agencies in infectious diseases are accustomed. Such an approach will require high levels of sustained funding of networks of research groups (ideally at least for 10 years). The approach will require collaborative research groups that follow a large number of well-defined patients over long periods. Success will depend on involvement of researchers highly skilled in clinical and epidemiologic investigation supported by laboratory personnel with proven expertise in detection of a wide spectrum of fastidious organisms. No single agent is likely to be the cause of chronic obstructive lung disease, asthma, or cancer; rather a number of infectious agents are likely to have this potential, hence the need for studies of large numbers of patients. Because the infecting agent may only be present in the very early stages of disease followed by an inflammatory response, different stages of disease need to be studied. A critical component of the investigative approach will be the ability to determine the genetic background and immune response of the patients.

The randomized, controlled clinical trial provides a scientific experiment that conforms to the standard model of biomedical research and is undoubtedly the best theoretical approach to evaluating any new therapy (110,111). Antibiotic treatment trials are commonly used to prove an infectious etiology. While clinical trials are at their best in evaluating the efficacy of therapies for acute diseases, clinical trials may not be the best approach for evaluating the efficacy of therapies for chronic diseases, most of which are likely to be complex, multifactorial illnesses in which behavioral and lifestyle factors play an important role. Some of the difficulties associated with this approach have already been discussed.

Well-defined, relevant animal models will be extremely important in elucidating the role of infectious agents in chronic inflammatory diseases. The animal studied should be the most genetically susceptible to the infecting agent and chronic infection. All too often inappropriate conclusions are based on use of a single strain of a single species. The value of using a naturally occurring disease with features that closely parallel those of the human disease cannot be overestimated.

As we attempt to prove the role of infection in chronic inflammatory diseases and cancer, the biggest challenge will be convincing peer review groups who establish research priorities and who facilitate funding decisions that these are not "fishing expeditions." Likewise, the challenge will be to convince journal editors that the findings are not merely coincidental. To make rapid progress we must keep an open mind and accept the likely possibility that fulfillment of Koch's postulates for infectious agents involved in chronic inflammatory diseases and cancer may not be possible.

Dr. Cassell is a recent past president of the American Society for Microbiology, a member of the National Institutes of Health Director's Advisory Committee, and a member of the Advisory Council of the National Institute of Allergy and Infectious Diseases of NIH. She was named to the original Board of Scientific Councilors of the National Center for Infectious Diseases, CDC, and is the immediate past chair of the board.

### References

1. Moscicki AB, Palefsky J, Gonzales J, Schoolnik GK. Human papillomavirus infection in sexually active adolescent females: prevalence and risk factors. Pediatr Res 1990;28:507-13.
2. Rous P. Transmission of a malignant new growth by means of a cell-free filtrate. JAMA 1911;56:198.
3. Cassell GH, Cole BC. Mycoplasmas as agents of human disease. N Engl J Med 1981;304:80-9.
4. Lindsey JR, Baker HJ, Overcash RG, Cassell GH, Hunt CE. Murine chronic respiratory disease: significance as a research complication and experimental production with *Mycoplasma pulmonis*. Am J Pathol 1971;64:675-708.
5. Hektoen L. Observations on pulmonary infections in rats. Transactions of the Chicago Pathology Society 1015-1918;10:105-8.
6. Nelson JB. Infectious catarrh of the albino rat. I. Experimental transmission in relation to the role of *Actinobacillus muris*. II. The causal relation of cocobacilliform bodies. J Exp Med 1940;72:645-54, 666-667.

7. Cassell GH, Lindsey JR, Baker HJ. Mycoplasmal and rickettsial diseases. In: Baker HJ, Lindsey JR, Weisbroth SH, editors. The laboratory rat, Vol. I. New York: Academic Press; 1979. p. 243-69.
8. Cassell GH, Lindsey JR, Overcash RG, Baker HJ. Murine mycoplasma respiratory disease. Ann N Y Acad Sci 1973;225:395-412.
9. Davis JK, Parker RF, White H, Dziedzic D, Taylor G, Davidson MK, et al. Strain differences in susceptibility to murine respiratory mycoplasmosis in C57BL/6 and C3H/HeN mice. Infect Immun 1985;50:647-54.
10. Davis JK, Thorp RB, Maddox PA, Brown MB, Cassell GH. Murine respiratory mycoplasmosis in F344 and LEW rats: evolution of lesions and lung lymphoid cell populations. Infect Immun 1982;36:720-9.
11. Cartner SC, Simecka JW, Briles DE, Cassell GH, Lindsey JR. Resistance to mycoplasmal lung disease in mice is a complex genetic trait. Infect Immun 1996;64:5326-31.
12. Simecka JW, Davis JK, Davidson MK, Ross SE, Städtlaender CTK-H, Cassell GH. Mycoplasma diseases of animals. In: Maniloff J, McElhaney R, Finch L, Baseman J, editors. Mycoplasmas: molecular biology and pathogenesis. Washington: American Society of Microbiology; 1992. p. 391-415.
13. Ross SF, Simecka JW, Gambill GP, Davis JK, Cassell GH. *Mycoplasma pulmonis* possesses a novel chemattractant for B lymphocytes. Infect Immun 1992;60:669,674.
14. O'Brodovich HM, Mellins RB. Bronchopulmonary dysplasia. Unresolved neonatal acute lung injury. Am Rev Respr Dis 1985;132:694-709.
15. Saigal S, Rosenbaum P, Stoskopf B, Sinclair JC. Outcome in infants 501-1000 gm birth weight delivered to residents of the McMaster Health Region. J Pediatr 1984;105:969-76.
16. Naeve RL, Dellinger WS, Blanc WA. Fetal and maternal features of antenatal bacterial infections. J Pediatr 1971;79:733-9.
17. Dennehy PH. Respiratory infections in the newborn. Clin Perinatol 1987;14:667-82.
18. Tafari N, Ross S, Naeye RL. Mycoplasma 'T' strains and perinatal death. Lancet 1976;1:108-9.
19. Cassell GH, Waites KB, Crouse DT, Rudd PT, Canupp KC, Stagno S, et al. Association of *Ureaplasma urealyticum* infection of the lower respiratory tract with chronic lung disease and death in very-low-birthweight infants. Lancet 1988;2:240-5.
20. Quinn PA, Gillan JE, Markestad T, St. John MA, Daneman A, Lie KI, et al. Intrauterine infection with *Ureaplasma urealyticum* as a cause of fatal neonatal pneumonia. Pediatr Infect Dis 1985;4:538-43.
21. Gray DJ, Robinson HB, Malone J. Adverse outcome in pregnancy following amniotic fluid isolation of *Ureaplasma urealyticum.* Prenat Diagn 1992;12:111-7.
22. Waites KB, Crouse DT, Philips JB III, Canupp KC, Cassell GH. Ureaplasmal pneumonia and sepsis associated with persistent pulmonary hypertension of the newborn. Pediatrics 1989;83:79-85.
23. Brus F, van Waarde WM, Schoots C, Oetomo SB. Fetal ureaplasmal pneumonia and sepsis in a newborn infant. Eur J Pediatr 1991;150:782-3.
24. Cassell GH, Waites KB, Gibbs RS, Davis JK. The role of *Ureaplasma urealyticum* in amnionitis. Pediatr Infect Dis 1986;5:S247-52.
25. Rudd PT, Carrington D. A prospective study of chlamydial, mycoplasmal and viral infections in a neonatal intensive care unit. Arch Dis Child 1985;59:120-5.
26. Taylor-Robinson D, Furr PM, Liberman MM. The occurrence of genital mycoplasmas in babies with and without respiratory diseases. Acta Paediatrica Scandinavica 1984;73:383-1984.
27. Ollikainen J, Heikkaniemi H, Korppi M, Sarkkinen H, Heinonen K. *Ureaplasma urealyticum* infection associated with acute respiratory insufficiency and death in premature infants. J Pediatr 1993;122:756-60.
28. Rudd PT, Cassell GH, Waites KB, Davis JK, Duffy LB. *Ureaplasma urealyticum* pneumonia: experimental production and demonstration of age-related susceptibility. Infect Immun 1989;57:918-25.
29. Walsh WF, Butler J, Coalson J, Hensley D, Cassell GH, Delemos RA. A primate model of *Ureaplasma urealyticum* infection in the premature infant with hyaline membrane disease. Clin Infect Dis 1993;17:S158-62.
30. Crouse DT, Odrezin GT, Cutter GR, Reese JM, Hamrick WB, Waites KB, et al. Radiographic changes associated with tracheal isolation of *Ureaplasma urealyticum* from neonates. Clin Infect Dis 1993;17:S122-30.
31. Sidiropoulos D, Herrmann U, Morell A. Transplacental passage of intravenous immunoglobulin in the last trimester of pregnancy. J Pediatr 1986;109:505-8.
32. Cassell GH. The pathogenic potential of mycoplasmas: *Mycoplasma pulmonis* as a model. Derrick Edward Award Lecture. Rev Infect Dis 1982;4:S18-34.
33. Cassell GH, Waites KB, Crouse DT. Mycoplasmal infections. In: Remington JS, Klein JO, editors. Infectious diseases of the fetus and newborn infant. Philadelphia (PA): W.B. Saunders Co.; 1994. p. 619-55.
34. Dyke MP, Grauaug A, Kohan R, Ott K, Andrews R. *Ureaplasma urealyticum* in a neonatal intensive care population. J Paediatr Child Health 1993;29:295-7.
35. Saxen H, Hakkarainen K, Pohjavuori M. Chronic lung disease of preterm infants in Finland is not associated with *Ureaplasma urealyticum* colonization. Acta Paediatr 1993;82:198-201.
36. Valencia GB, Banzon F, Cummings M. *Mycoplasma hominis* and *Ureaplasma urealyticum* in neonates with suspected infection. Pediatr Infect Dis J 1993;12:571-3.
37. Crouse DT, Cassell GH, Waites KB, Foster JM, Cassady G. Hyperoxia potentiates *Ureaplasma urealyticum* pneumonia in newborn mice. Infect Immun 1990;58:3487-93.
38. Stancombe BB, Walsh WF, Derdak S, Dixon P, Hensley D. Induction of human neonatal pulmonary fibroblast cytokines by hyperoxia and *Ureaplasma urealyticum.* Clin Infect Dis 1993;17:S154-7.
39. Payne NR, Steinberg S, Ackerman P, Cheenka BA, Sane SM, Anderson KT, et al. New prospective studies of the association of *Ureaplasma urealyticum* colonization and chronic lung disease. Clin Infect Dis 1993;17:S117-21.
40. Cassell GH, Davis RO, Waites KB, Brown MB, Marriott PA, Stagno S, et al. Isolation of *Mycoplasma hominis* and *Ureaplasma urealyticum* from amniotic fluid at 16-20 weeks gestation: potential effect on outcome of pregnancy. Sex Transm Dis 1983;10:294-302.

41. Parker RF, Davis JK, Cassell GH, White H, Dziedzic D, Blalock DK, et al. Short-term exposure to nitrogen dioxide enhances susceptibility to murine respiratory mycoplasmosis and decreases intrapulmonary killing of *Mycoplasma pulmonis*. Am Rev Respir Dis 1989;140:502-12.
42. National Heart, Lung and Blood Institute Data Fact Sheet, Asthma Statistics, May, 1992. Washington: National Institutes of Health; 1992.
43. Rao M, Kravath R, Abadco D, Arden J, Steiner P. Childhood asthma mortality: the Brooklyn experience and a brief review. J Assoc Acad Minor Phys 1991;2:127-30.
44. Sterk PJ. Virus-induced airway hyperresponsiveness in man. Eur Respir J 1993;6:894-902.
45. Cassell GH, Clyde WA, Davis JK. *Mycoplasmal* respiratory infections. In: Razin S, Tully JG, editors. The *Mycoplasmas*. New York: Academic Press; 1985. p. 66-106.
46. Shimuzu T, Mochizuki H, Kato M, Shjigeta M, Morikawa A, Hori T. Immunoglobulin levels, number of eosinophils in the peripheral blood and bronchial hypersensitivity in children with *Mycoplasma pneumoniae* pneumonia. Japanese Journal of Allergology 1991;40:21-7.
47. Sabato AR, Martin AJ, Marmion BP, Kok TW, Cooper DM. *Mycoplasma pneumoniae*: acute illness, antibiotics, and subsequent pulmonary function. Arch Dis Child 1984;59:1034-7.
48. Seggev JS, Lis I, Siman-Tov S, Gutman R, Abu-Samara H, Bouchey H, et al. *Mycoplasma pneumoniae* is a frequent cause of exacerbation of bronchial asthma in adults. Annals of Allergy 1986;57:262-5.
49. Yano T, Ichikawa Y, Komatu S, Arai S, Oizumi K. Association of *Mycoplasma pneumoniae* antigen with initial onset of bronchial asthma. Am J Respir Crit Care Med 1994;149:1348-53.
50. Henderson FW, Clyde Jr WA, Collier AM, Denny FW, Senior RJ, Sheaffer CI, et al. The etiologic and epidemiologic spectrum of bronchiolitis in pediatric practice. J Pediatr 1979;95:183-90.
51. Grayston JT. Infections caused by *Chlamydia pneumoniae* strain TWAR. Clin Infect Dis 1992;15:757-63.
52. Hahn DL, Dodge RW, Golubjatnikov R. Association of *Chlamydia pneumoniae* (TWAR) infection with wheezing, asthmatic bronchitis and adult-onset asthma. JAMA 1991;266:225-30.
53. Emre U, Roblin PM, Gelling M, Dumornay W, Rao M, Hammerschlag MR, et al. The association of *Chlamydia pneumoniae* infection and reactive airway disease in children. Archives of Pediatric Medicine 1994;148:727-32.
54. Hammerschlag MR, Chirgwin K, Roblin PM. Persistent infection with *Chlamydia pneumoniae* following acute respiratory illness. Clin Infect Dis 1992;14:178-222.
55. Kraft M, Cassell GH, Henson JE, Watson H, Williamson J, Marmion BP, et al. Detection of *Mycoplasma pneumoniae* in the airways of adults with chronic asthma. Am J Resp Crit Care Med. In press 1998.
56. Allegra L, Blasi F, Centanni S, Cosentini R, Denti F, Raccanelli R, et al. Acute exacerbations of asthma in adults: role of *Chlamydia pneumoniae* infection. Eur Respir J 1994;7:2165-8.
57. Hahn DL, Golubjatnikov R. Asthma and Chlamydial infection: a case series. J Fam Pract 1994;38:589-95.
58. Block S, Hedrick J, Hammerschlag AR, Craft JC. *Mycoplasma pneumoniae* and *Chlamydia pneumoniae* in pediatric community-acquired pneumonia: comparative safety and efficacy of clarithromycin vs. erythromycin. Pediatr Infect Dis 1995;14:471-7.
59. Harris JAS, Kolokathis A, Campbell M, Cassell GH, Hammerschlag MR. Safety and efficacy of azithromycin treatment of community acquired pneumonia in children. Pediatr Infect Dis J. In press 1998.
60. Giron JA, Lange M, Baseman JB. Adherence, fibronectin, binding, and induction of cytoskeleton reorganization in cultured human cells by *Mycoplasma penetrans*. Infect Immun 1996;64:197-208.
61. Wise KS, Cassell GH, Acton RT. Selective association of murine T lymphoblastoid cell surface alloantigens with *Mycoplasma hyorhinis*. Proc Natl Acad Sci U S A 1978;75:4479-83.
62. Marmion BP, Williamson J, Worswick PA, Kok TW, Harris RJ. Experience with newer techniques for the laboratory detection of *Mycoplasma pneumoniae* infection: Adelaide, 1978-1991. Clin Infect Dis 1993;17:S90-9.
63. Cassell GH, Drnec J, Waites KB, Pate MS, Duffy LB, Watson HL, et al. Efficacy of clarithromycin against *Mycoplasma pneumoniae*. J Antimicrob Chemother 1991;27:47-59.
64. Gray GC, Duffy LB, Paver RJ, Putnam SD, Reynolds RJ, Cassell GH. *Mycoplasma pneumoniae*: a frequent cause of pneumonia among U.S. marines in southern California. Mil Med 1997;162:524-6.
65. Osler W. Diseases of the arteries. In: Osler W, editor. Modern medicine: its practice and theory. Philadelphia (PA): Lea & Febiger; 1908. p. 429-47.
66. Fabricant CG, Fabricant J, Litrenta MM, Minick CR. Virus-induced atherosclerosis. J Exp Med 1978:335-40.
67. Danesh J, Collins R, Peto R. Chronic infections and coronary heart disease: is there a link? Lancet 1997;350:430-6.
68. Patel P, Mendall MA, Carrington D, Strachan DP, Leatham E, Molineaux N, et al. Association of *Helicobacter pylori* and *Chlamydia pneumoniae* infections with coronary heart disease and cardiovascular risk factors. BMJ 1995;311:711-4.
69. Melnick JL, Adam E, Debakey ME. Cytomegalovirus and atherosclerosis. Eur Heart J 1993;14:30-8.
70. Beck J, Garcia R, Heiss G, Vokonas PS, Offenbacher S. Periodontal disease and cardiovascular disease. J Periodontol 1996;67:1123-37.
71. File TM, Bartlett JG, Cassell GH, Gaydos CA, Grayston JT, Hammerschlag MR, et al. The importance of *Chlamydia pneumoniae* as a pathogen: the 1996 consensus conference on *Chlamydia pneumoniae* infections. Infec Dis Clin Prac1997;6:S28-31.
72. Kuo C, Shor A, Campbell L, Fukushi H, Patton DL, Grayston JT. Demonstration of *Chlamydia pneumoniae* in atherosclerotic lesions of coronary arteries. J Infect Dis 1993;167:841-9.
73. Kuo CC, Grayston JT, Campbell LA, Goo YA, Wissler RW, Benditt EP. *Chlamydia pneumoniae* (TWAR) in coronary arteries of young adults (15-34 years old). Proc Natl Acad Sci U S A 1995;92:6911-4.
74. Campbell LA, O'Brien ER, Capuccio AL, Kuo C-C, Wang S-P, Stewart D. Detection of *Chlamydia pneumoniae* TWAR in human coronary arterectomy tissues. J Infect Dis 1995;172:585-8.
75. Ong G, Thomas BJ, Mansfield AO, Davidson BR, Taylor-Robinson D. Detection and widespread distribution of *Chlamydia pneumoniae* in the vascular system and its possible implications. J Clin Pathol 1996;49:102-6.

76. Jackson LA, Lee AC, Cho-Chou Kuo, Rodriquez DI, Lee A, Grayston JT. Isolation of *Chlamydia pneumoniae* from a carotid endarterectomy specimen. J Infect Dis 1997;176:292-5.
77. Blasi F, Denti F, Erba M. Detection of *Chlamydia pneumoniae* but not *Helicobacter pylori* in atherosclerotic plaques of aortic aneurysms. J Clin Microbiol 1996;34:2766-9.
78. Muhlestein JB, Hammond EH, Carlquist JF, Radicke E, Thomson MJ, Karagounis LA, et al. Increased incidence of *Chlamydia* species within the coronary arteries of patients with symptomatic atherosclerotic versus other forms of cardiovascular disease. J Am Coll Cardiol 1996;27:1555-61.
79. Kuo CC, Jackson LA, Campbell LA, Grayston JT. *Chlamydia pneumoniae* (TWAR). Clin Microbiol Rev 1995;8:451-61.
80. Yang ZP, Kuo CC, Grayston JT. Systemic dissemination of *Chlamydia pneumoniae* following intranasal inoculation in mice. J Infect Dis 1995;171:736-8.
81. Fong IW, Chiu B, Viira E, Fong MW, Jang D, Mahony J. Rabbit model for *Chlamydia pneumoniae* infection. J Clin Microbiol 1997;35:48-52.
82. Laitinen K, Laurila A, Pyhala L, Leinonen M, Saikku P. *Chlamydia pneumoniae* infection induces inflammatory changes in the aortas of rabbits. Infect Immun 1997;65:4832-5.
83. Kaukoranta-Tolvanen SS, Teppo AM, Laitinen K, Linnavuori K, Leinonen M. Growth of *Chlamydia pneumoniae* in cultured human peripheral blood mononuclear cells and induction of a cytokine response. Microb Pathog 1996;21:215-21.
84. Molestina R, Miller RD, Summersgill JT, Ramirez. *Chlamydia pneumoniae* stimulates secretion of chemokines and adhesion molecules in human endothelial cells. In: Abstracts of the 96th General Meeting of the American Society for Microbiology 1996. Washington: American Society for Microbiology; 1996. Abstract No. 243.
85. Nurmenen M, Leinonen M, Saikku P, Makela PH. The genus-specific antigen of *Chlamydia*: resemblance to the lipopolysaccharide of enteric bacteria. Science 1988;220:1279-81.
86. Saikku P, Leinonen M, Tenkanen L, Linnanmaki E, Ekman MR, Manninen V, et al. Chronic *Chlamydia pneumoniae* infection as a risk factor for coronary heart disease in the Helsinki heart study. Ann Intern Med 1992;116:273-8.
87. Gupta S, Leatham EW, Carrington D, Mendall MA, Kaski JC, Camm J. Elevated *Chlamydia pneumoniae* antibodies, cardiovascular events, and azithromycin in male survivors of myocardial infarction. Circulation 1997;96:404-7.
88. Gurfinkel E, Bozovich G, Daroca A, Beck E, Mautner B, for the ROXIS Study Group. Randomised trial of roxithromycin in non-Q-wave coronary syndromes: ROXIS pilot study. Lancet 1997;350:404-7.
89. Liddy P, Egan D, Skarlartos S. Roles of infectious agents in atherosclerosis and restenosis. Circulation 1997;96:4095-103.
90. Kadota JL. Non antibiotic effects of antibiotics. J Clin Micro Infect 1996;1:220-2.
91. Labro MT. Intracellular bioactivity of macrolides [suppl]. J Clin Micro Infect 1996:1:24-30.
92. Agen C, Danesi R, Blandizzi C. Macrolide antibiotics as antiinflammatory agents:roxithromycin in an unexpected role. Agents Actions 1993;38:85-90.
93. Kita E, Sawaki M, Mikasa K. Alterations of host response by long term treatment of roxithromycin. J Antimicrob Chemother 1993;32:285-94.
94. Pisani P, Parkin DM, Munoz, Ferlay J. Cancer and infectiou: Estimates of the attributable fraction in 1990. Can Epidemiol Biomarkers Prevent 1997;6:387-400.
95. Infectious diseases and cancer. 1996. In: The World Health Report 1996. Fighting disease fostering development. Geneva: World Health Organization; 1996. p. 59-62.
96. Parsonnet J. *Helicobacter pylori*. Infect Dis Clinics North Amer 1998;12:185-97.
97. Marshall BJ. History of the discovery of *C. pylori*. In Blaser MJ, editor. *Campylobacter pylori* in gastritis and peptic ulcer disease. New York: Igaku-Shoin; 1989. p. 7-23.
98. Moller H, Heseltine E, Vaqinio. Working group report on schistosomes, liver flukes, and *Helicobacter pylori*. Int J Cancer 1995;60:587-9.
99. NIH Consensus Conference. *Helicobacter pylori* in peptic ulcer disease. JAMA 1994;272:65-9.
100. Xiang Z, Censini S, Bayeli PF, Telford JL, Figura N, Rappuoli R, et al. Analysis of expression of CagA and VacA virulence factors in 43 strains of *Helicobacter pylori* reveals that clinical isolates can be divided into two major types and that CagA is not necessary for expression of the vacuolating cytotoxin. Infect Immun 1995;63:94-8.
101. Blaser MJ, Perez-Perez GI, Kleanthous H, Cover TL, Peek RM, Chyou PH. Infection with *Helicobacter pylori* strains possessing cagA is associated with an increased risk of developing adenocarcinoma of the stomach. Cancer Res 1995;55:2111-5.
102. Chen CJ, Lai MS, Hsu HM, Wu TC, Kong MS, Liang DC, et al. Universal hepatitis B vaccination in Taiwan and the incidence of hepatocellular carcinoma in children. Taiwan Childhood Hepatoma Study Group. N Engl J Med 1997;336:1855-9.
103. Hepatitis viruses. Monographs on the evaluation of carcinogenic risks to humans. Lyon, France: IARC; 1994. IARC Scientific Publ No. 59.
104. Chuang WL, Chang WY, Lu SN, Su WP, Lin ZY, Chen SC, et al. The role of hepatitis B and C viruses in hepatocellular carcinoma in a hepatitis B endemic area. A case-control study. Cancer 1992;69:2052-4.
105. Human papillomaviruses. Monographs on the evaluation of carcinogenic risks to humans. Lyon, France: IARC; 1995. IARC Scientific Publ No. 64.
106. Vousden KH, Farrel PJ. Viruses and human cancer. Br Med Bull 1994;3:580-1.
107. Morris JDH, Eddleston ALWF, Crook T. Viral infection and cancer. Lancet 1995;346:754-8.
108. Ohshima H, Bartsch H. Chronic infections and inflammatory processes as cancer risk factors: possible role of nitric oxide in carcinogenesis. Mutat Res 1994;305:253-64.
109. Mackowiak PA. Microbial latency. Rev Infect Dis 1984;6:649-67.
110. Pincus T. Rheumatoid arthritis: disappointing long-term outcomes despite successful short-term clinical trials. J Clin Epidemiol 1988;41:1037.
111. Feinstein AR. An additional basic science for clinical medicine: II. The limitations of randomized trials. Ann Intern Med 1983;544.
